# Current evidence and strategies for preventing tumor recurrence following thermal ablation of papillary thyroid carcinoma

**DOI:** 10.1186/s40644-025-00908-7

**Published:** 2025-07-09

**Authors:** Ru Li, Luyang Yang, Ming Xu, Baofeng Wu, Qinhao Liu, Qin An, Yuchen Sun, Yi Zhang, Yunfeng Liu

**Affiliations:** 1 Department of Endocrinology, First Hospital of Shanxi Medical University, Taiyuan, China; 2 First Clinical Medical College, Shanxi Medical University, Taiyuan, China; 3 Department of Geriatrics, Changzhi People’s Hospital, Changzhi, China; 4 Department of Pharmacology, Shanxi Medical University, Taiyuan, China; 5 Clinical Research Center for Metabolic Diseases of Shanxi Medical University, Taiyuan, China

**Keywords:** Ablation techniques, Papillary thyroid carcinoma, Tumor residual, Lymph node metastasis

## Abstract

**Background:**

The incidence of papillary thyroid carcinoma (PTC) has been increasing, and thermal ablation has emerged as a minimally invasive alternative to surgery for low-risk cases. However, post-ablation tumor progression remains a significant clinical challenge.

**Methods:**

This review synthesizes existing literature on tumor progression after thermal ablation for PTC, analyzing potential causes and evaluating preventive strategies at different diagnostic and treatment stages.

**Results:**

Current research reports indicate that the probability of disease progression following thermal ablation for PTMC ranges from 1.25 to 7.7%, a rate comparable to that of surgical management. Nodules exceeding 10 mm in diameter are associated with a higher risk of post-procedural progression. However, pathological evidence supporting these findings remains limited. Risk factors such as suboptimal patient selection and tumor proximity to critical structures further influence outcomes. Improved imaging guidance, standardized protocols, and stringent follow-up may reduce these complications.

**Conclusion:**

When these recommendations are followed, thermal ablation for PTMC achieves effective reduction in tumor progression risk and represents a viable alternative for appropriately selected patients. However, expansion of its indications requires further robust evidence from large-scale, pathology-based studies.

## Introduction

Papillary thyroid carcinoma (PTC) is the most prevalent form of thyroid cancer, accounting for approximately 80% of all cases [1]. Owing to the advancement of ultrasound (US) technology and the widespread adoption of US-guided fine needle biopsy, the prevalence has been increasing annually. Surgical resection remains the main and preferred treatment option; but it inevitably results in postoperative complications such as hypothyroidism, tissue damage, and cervical scarring. For low-risk papillary thyroid microcarcinoma (PTMC), active surveillance is recommended as an alternative approach by multiple authoritative guidelines [2]. However, because of anxiety and the potential for tumor progression, it may not be the optimal choice for all patients. In recent years, thermal ablation techniques, including radiofrequency ablation (RFA), microwave ablation (MWA), and laser ablation, have been increasingly utilized in the treatment of PTC, particularly for PTMC [3]. Specifically, under the guidance of US, local high temperature is generated within the tumor tissue, resulting in coagulation necrosis of cancer cells. This technique offers several advantages, such as being minimally invasive, easily tolerated by the patient, less tissue damage, and high repeatability.

Currently, the safety and efficacy of thermal ablation in PTMC patients have been thoroughly validated. Multiple domestic and international guidelines and consensus also recommend thermal ablation as a viable treatment option for patients who are not candidates for surgery due to intolerance or personal refusal [4–6]. Numerous studies have explored the application of thermal ablation technology for off-label treatment of PTC. Although thermal ablation can reduce the size of the visible tumour significantly, or even make it disappear completely, it is uncertain whether the tumour area has been completely eradicated. Several studies have documented that patients with PTC after thermal ablation may experience disease progression, including local residuals, tumor recurrence, and lymph node metastasis (LNM). Consequently, these patients frequently require secondary ablation or a transition to surgical intervention, which significantly impairs their quality of life and restricts the broader application of thermal ablation technology in PTC treatment. From a microscopic perspective, sublethal thermal stimulation of residual PTC cells after ablation can enhance their clonogenic potential, migratory capacity, and invasive ability [7], which further reflects the importance of achieving complete ablation.

To date, there remains a lack of consensus regarding the standardization of thermal ablation for the treatment of PTC in order to prevent tumor progression. Indeed, this issue pervades the entire spectrum of preoperative assessment, intraoperative procedures, and postoperative follow-up. In this review, we systematically analyzed the existing literature to summarise the data and underlying mechanisms of disease progression. Furthermore, we propose targeted improvement strategies aimed at guiding clinical practice in the application of thermal ablation of PTC.

## Clinical progression data following thermal ablation treatment for PTC

Concerning the incidence of disease progression in PTC patients treated with thermal ablation, the findings exhibit variability across different populations and studies. In a large prospective cohort study of 1278 low-risk PTMC patients undergoing MWA treatment, 6 patients (0.47%) developed local tumor progression and 10 patients (0.78%) developed cervical LNM during a mean follow-up period of 34.57 months [8]. A retrospective study compared the clinical outcomes of RFA and lobectomy in the treatment of PTMC. The results indicated that after a median follow-up of 48.1 months, the incidence rates of local tumor residual, tumor recurrence, and LNM in the RFA group were 0.2% (1/424), 2.4% (10/424), and 0.9% (4/424), respectively. The rate of tumor progression was comparable between the RFA and lobectomy groups [9]. A meta-analysis encompassed 1,770 patients who underwent RFA of PTMC. Following a mean follow-up period of 33 months, the overall tumor progression rate was 1.5%, including 7 cases of local residual tumor (0.4%), 15 cases of new-onset PTMC (0.9%), and 4 cases of LNM (0.2%) [10]. During the more than 10-year follow-up of 65 low-risk PTMC patients treated with RFA, no local tumor progression or metastasis was observed, but 7.7% of the patients developed a new primary thyroid carcinoma [11].

Several studies have explored the application of thermal ablation techniques in PTC with diameters exceeding 10 mm. Despite the majority of studies demonstrating satisfactory clinical efficacy, the issue of disease persistence or progression following ablation therapy remains a significant concern that cannot be overlooked. A large-scale study conducted by Li X et al. assessed the long-term prognosis of 1,613 PTC patients treated with RFA. With a mean follow-up duration of 58.5 months, 27 (1.7%) patients found tumours at the ablation site, while 42 (2.6%) patients had tumour recurrence. Compared to patients with stage T1a tumors (diameter ≤ 1 cm), those with stage T1b tumors (1 cm < diameter ≤ 2 cm) exhibited a higher likelihood of local tumor progression (8.8% vs. 3.7%) and tumor persistence (5.2% vs. 1.2%). In addition, tumors located within 2 mm of the capsule or trachea, as well as multifocal tumors, were identified as independent risk factors for local tumour progression [12]. According to findings from a large-scale study, these were no significant differences in the clinical outcomes between RFA and MWA in the treatment of T1N0M0 PTC [13]. A multicenter study reporting 10-year outcomes of thermal ablation for T1N0M0 PTC demonstrated local progression in 11 out of 179 patients (6.1%). It included 4 cases with LNM, 6 cases with de novo tumors, and 1 case with persistent disease [14]. Fei YL et al. recently conducted a comprehensive evaluation of the efficacy and safety of thermal ablation in 34 patients with isolated low-risk T2N0M0 PTC, where the tumor diameter was greater than 2 cm but no more than 4 cm. After one year of follow-up, one patient experienced local tumor progression and another developed new lesions [15]. Nonetheless, given the limited sample size and the relatively brief follow-up duration of this study, additional research is warranted.

However, the majority of clinical studies predominantly utilize conventional US or contrast-enhanced ultrasound (CEUS) for follow-up examinations. Fine-needle aspiration biopsy (FNAB) or core needle biopsy (CNB) is conducted only when there is a suspicion of tumor recurrence or LNM. Because of the limitations of US in accurately assessing the microscopic changes of tumors, it may underestimate the incidence of tumor progression. A 5-year histological follow-up of patients treated with RFA for T1N0M0 PTC revealed that tumor persistence or recurrence occurred in 10 (2.9%) out of 341 patients with stage T1a, compared to 12.2% of 41 patients with stage T1b. It also indicated that tumor size and subcapsular location were significant risk factors [16]. Ma B et al. reported the outcomes of thermal ablation in 12 cases of lesions, with an average size of 1.3 ± 0.7 cm. Histopathological examination confirmed residual tumor cells in all cases, and cervical LNM was identified in 8 cases (66.7%) [17]. Ding M et al. also reported a cohort of 12 patients who underwent surgical intervention after MWA therapy. Residual tumors were identified in 2 cases, new lesion was detected in 3 cases, and cervical LNM was observed in 7 cases [18]. As these subsequent studies specifically enrolled patients with clinical suspected or confirmed tumor progression, they cannot be used to assess the probability of tumor progression following thermal ablation for PTC. Therefore, more prospective studies incorporating histological follow-up are essential to accurately evaluate the efficacy of this treatment modality.

## Causes of disease progression after thermal ablation of PTC

### Limitations of imaging modalities in the assessment of latent lesions

High-resolution US is the primary imaging modality for evaluating thyroid nodules. It not only provides detailed characterization of nodule features but also assesses potential cervical LNM. CEUS helps assess the ablation extent and confirm the achievement of complete ablation. Due to the limitations of US in detecting microscopic tumor cells, occult carcinoma in situ and LNM may not be readily identified, which may lead to disease progression after ablation. Furthermore, ultrasonography of the mediastinum, clavicle, retropharyngeal, and parapharyngeal regions is challenging, which increases the risk of missed diagnoses of cervical lymph nodes. A study of 2,424 low-risk PTMC patients who underwent surgical resection revealed that 11.8% exhibited extrathyroidal extension and 31.3% had LNM [19]. Another study examining low-risk PTMC cases deemed suitable for thermal ablation demonstrated that 34.7% of patients had occult lesions beyond the target tumor, encompassing ipsilateral or contralateral occult cancers as well as LNM [20]. Sun W et al. reported on 21 PTC patients who underwent surgical treatment for residual lesions following RFA therapy. Postoperative pathology found that 23.8% (5/21) of patients with LNM had a staging of CN0 [21]. The common risk factors for PTMC with LNM include male, age, lesion size, multifocal tumors, and extrathyroidal extension [22–24]. Compared to PTMC, the likelihood of LNM was significantly higher in cases where the tumor diameter exceeded 1 cm [25].

Computed tomography (CT) and magnetic resonance imaging (MRI) can complement the limitations of US; however, neither CT nor MRI alone surpasses US in certain diagnostic capabilities [26, 27]. A meta-analysis came to a conclusion that the combination of cervical CT and US is the optimal approach for detecting cervical LNM in PTC [28]. Nevertheless, using contrast-enhanced CT requires attention to the risk of postoperative thyroid dysfunction [29]. Lymphatic contrast-enhanced ultrasound (LCEUS) is capable of identifying LNM through distinct enhancement patterns. Preliminary studies with limited sample sizes have proved that LCEUS exhibits superior diagnostic accuracy compared to conventional US for detecting LNM smaller than 1 cm [30]. Unfortunately, its application in PTC remains in the early stages of exploration. In addition, several studies have employed clinical characteristics, US parameters, and multi-omics approaches to develop models for predicting cervical LNM in PTC [31, 32]. However, none of these models have yet achieved widespread clinical application.

### Uncertainty associated with FNAB for pathological diagnosis

FNAB is the primary diagnostic method for differentiating between benign and malignant thyroid nodules preoperatively. In contrast to conventional CNB, FNAB can only acquire diseased cells through fine needle aspiration, thereby limiting its ability to provide a detailed pathological classification of nodules. Because not all PTC are biologically inert and may exhibit varying degrees of biological aggressiveness, different types of thyroid cancer can lead to distinct prognoses. Besides, the results of FNAB are significantly influenced by cellular quantity. When nodules are small or exhibit calcification, acquiring adequate specimens via FNAB becomes more challenging, increasing the likelihood of false-negative results. In a study, the results of FNAB were inconsistent with postoperative pathology in 15.6% of patients, and all these nodules measured less than 1 cm in diameter [21].

FNAB molecular diagnosis can complement cytological analysis in the risk stratification of thyroid nodule malignancy. The BRAF V600E mutation is considered to be strongly associated with PTC. A meta-analysis involving 9,924 FNAB samples confirmed that the BRAF V600E mutation exhibits 100% specificity and 69% sensitivity in the diagnosis of PTC [33]. The role of BRAF V600E mutations in the PTC remains a subject of debate in the literature. Several researchers have posited that the BRAF V600E mutation may be regarded as a risk factor for LNM [34] and an independent predictor of long-term recurrence among PTC patients [35]. Conversely, other studies suggest that it is not correlated with adverse prognosis in low-risk PTC cases [36]. Dong SY et al. proposed that the clinical significance of BRAF V600E mutations necessitates differentiation based on histological subtypes and preoperative central lymph node status [37]. Therefore, whether to include the BRAF V600E mutation as a criterion for thermal ablation remains inconclusive.

In addition to the aforementioned genetic alteration, RAS mutation, RET rearrangement and telomerase reverse transcriptase (TERT) promoter mutation are also frequently observed in PTC. But no single factor can adequately stratify the risk levels of PTC. RAS mutation is more likely to be associated with the follicular variant of PTC. In comparison to BRAF-positive malignancies, RAS-positive malignancies demonstrate a lower degree of aggressiveness [38]. RET rearrangement-positive carcinomas are linked to a significantly higher incidence of LNM [39], with an incidence rate of 75.2% [40]. A meta-analysis concluded TERT promoter mutation-positive PTC was significantly more likely to exhibit an aggressive clinicopathological characteristics [41]. There is evidence that multiple co-existing genetic alterations significantly accelerate disease progression, so it is important to provide a comprehensive assay [42, 43].

For suspected malignant LNM, cytological examination can provide a diagnostic sensitivity ranging from 66–95% [44]. However, for cystic lymph nodes, as many as 28.5% of cases cannot be definitively diagnosed due to the confounding effects of lymphocyte infiltration or necrotic cells [45]. Simultaneously, cytological diagnosis can be significantly influenced by the subjective factors of the pathologist. The detection of thyroglobulin in fine needle aspiration (FNA-Tg) was initially proposed by Pacini et al. in 1992 [46], and subsequent studies have demonstrated its efficacy in enhancing the sensitivity of cytological diagnosis [47–49]. But standardization of the eluent and critical values for FNA-Tg has yet to be achieved. A meta-analysis was conducted to compare the diagnostic efficacy of various methods for detecting LNM of thyroid cancer. Among invasive examination techniques, the combination of fine-needle aspiration cytology with FNA-Tg measurement showed superior diagnostic performance of 86.6% sensitivity and 85.8% specificity [50].

CNB demonstrated a significantly lower proportion of non-diagnostic and uncertain results compared to FNAB [51]. Due to its invasive nature and associated complications, it is not commonly employed as a pre-diagnostic method for thermal ablation of PTC. It is only recommended for patients with FNAB-undiagnosed thyroid nodules.

### Nodules in proximity to critical structures

The thyroid gland is situated in the anterior midline of the neck, with its isthmus overlying the trachea. The glandular lobes are adjacent to both the trachea and esophagus, while laterally they approach the major blood vessels of the neck. The parathyroid glands are attached posteriorly, and the recurrent laryngeal nerve is located within the tracheoesophageal groove posterior to the thyroid. In this confined space, in order to prevent thermal damage to surrounding structures, operators tend to opt for low-power and short-duration ablation, which may result in incomplete ablation. The “danger triangle” area comprises the dorsal edge of the thyroid gland, the lateral tracheal wall, and the anterior edge of the esophageal wall. When PTC tumors are present within the “danger triangle”, it can complicate technical success. Surprisingly, previous studies on PTC treated by thermal ablation near the “danger triangle” have not demonstrated significant local recurrence [52, 53]. It is important to note that the distance between the nodule and the tracheoesophageal groove has been identified as an independent risk factor for postoperative hoarseness [54].

### Heat sink effects

Blood flow can cool surrounding tissues via convective heat transfer and temperature gradients, which may result in a reduction of temperature during thermal ablation. This phenomenon is commonly referred to as heat sink effect. This effect hinders the attainment of lethal temperatures in tumor tissues surrounding blood vessels, thereby allowing tumor cells to persist. Since RFA utilizes electrical current to generate heat within tissues and is significantly influenced by tissue conductivity, the effect becomes particularly pronounced during RFA treatments. It is a critical factor contributing to incomplete ablation, which has been evidenced in the clinical application of RFA therapy for liver tumors [55]. A flow-dependent heat sink effect was also observed in a vitro model under thyroid-specific ablation conditions [56]. Therefore, it is reasonable to assume that the presence of excessive peripheral blood vessels or large vessels significantly contributes to treatment failure. Compared to other thermal ablation techniques, MWA is unaffected by electrical conductivity and can produce a broader thermal distribution within a shorter timeframe. As a result, it exhibits lower sensitivity to heat dissipation from blood vessels. Based on the results published, there was no significant difference in the local recurrence rate between T1N0M0 PTC treated with RFA and MWA [57]. Nevertheless, for patients with PTMC, the ablation time required for RFA is longer compared to MWA, and a greater volume of separated fluid is utilized [58].

### Needle track seeding

Percutaneous insertion of electrodes into malignant tumors may lead to the dissemination of tumor cells along the needle track. This phenomenon is predominantly observed in the thermal ablation treatment of liver cancer, while is less frequently reported in the thyroid cancer. In 2014, Lee CU et al. reported a case of a 19-year-old female who developed a neoplasm in the platysma muscle along the needle entry pathway, two years following RFA treatment of a thyroid nodule [59]. In 2017, Oddo et al. also documented a case involving a 74-year-old female patient who developed hypoechoic nodules in the median fascia of the external cervical thyroid gland 30 months following RFA treatment [60]. It is important to highlight that both patients presented with large tumors, and the postoperative pathological findings indicated an aggressive solid subtype of PTC or follicular carcinoma. To date, there have been no reported instances of needle tract seeding following the ablation of low-risk PTMC.

Several mechanisms are proposed to explain the dissemination of tumor cells: 1) Surviving tumor cells may adhere to the electrode during its retraction, while the internally cooled electrode may be insufficient for completely eradicating these cells. 2) Tumor cells may also be introduced into the needle tract as a result of intratumoral hemorrhaging. 3) During thermal ablation, tumor cells may be expelled into the needle tract due to the sudden increase in intratumoral pressure [61, 62]. Ablation of the needle tract following the completion of the procedure is an effective method to prevent potential tumor cell dissemination along the needle track [63].

## Strategies to prevent disease progression following thermal ablation in patients with PTC

In addition to tumor progression due to the above reasons, the influence of the operator’s experience and technical proficiency on ablation outcomes has been validated in benign nodules. Consequently, before the ablation of PTC, the operator should be proficient in US, FNAB and ablation of benign thyroid nodules [64]. On this basis, the following strategies can help reduce tumor progression.

### Rigorous preoperative assessment

Despite being included in treatment guidelines for PTMC in several countries as an alternative for low-risk patients who cannot tolerate or refuse surgery, thermal ablation comes with multiple stringent criteria. These include non-invasive subtypes, absence of multifocal lesions, and no evidence of lymph node or distant metastasis [4, 6, 65]. It requires a comprehensive assessment through imaging examination including high-resolution US, CEUS, CT and MRI. Furthermore, it imposes heightened demands on the precision of FNAB. For nodules with small diameters, calcification, or abundant vascularity, the number of punctures can be appropriately increased to enhance the likelihood of successful sampling. Influenced by the subjective factors, collaboration between experienced radiologists and pathologists across multiple disciplines is also essential.

For patients who do not meet the indications, it is essential to comprehensively inform them of all treatment options, including a detailed explanation of the advantages and disadvantages. Obtaining informed consent from patients is crucial, rather than solely recommending thermal ablation as the sole treatment option or overstating its benefits. Although numerous studies have assessed off-label efficacy and safety, concerns remain despite demonstrating promising results. This is primarily due to the absence of long-term prospective study designs and rigorous follow-up methodologies.

### Standardized intraoperative techniques

#### The trans-isthmic approach and alienate maneuver

The trans-isthmic approach involves inserting the ablation electrode into the target nodule via the isthmus and extending it laterally towards the neck when the nodule is located in the thyroid lobe. In addition to maintaining the electrode position with relative stability, this approach can continuously monitor the spatial relationship between the electrode, recurrent laryngeal nerve, and target nodule, so as to prevent potential thermal injury during the procedure. Furthermore, the presence of normal isthmus parenchyma between the target nodule and the electrode insertion site acts as a barrier, preventing the leakage of thermal ablation fluid into the perithyroid area. This not only reduces intraoperative pain but also avoids premature termination of the ablation procedure due to inadequate patient tolerance [66].

**Fig. 1:**
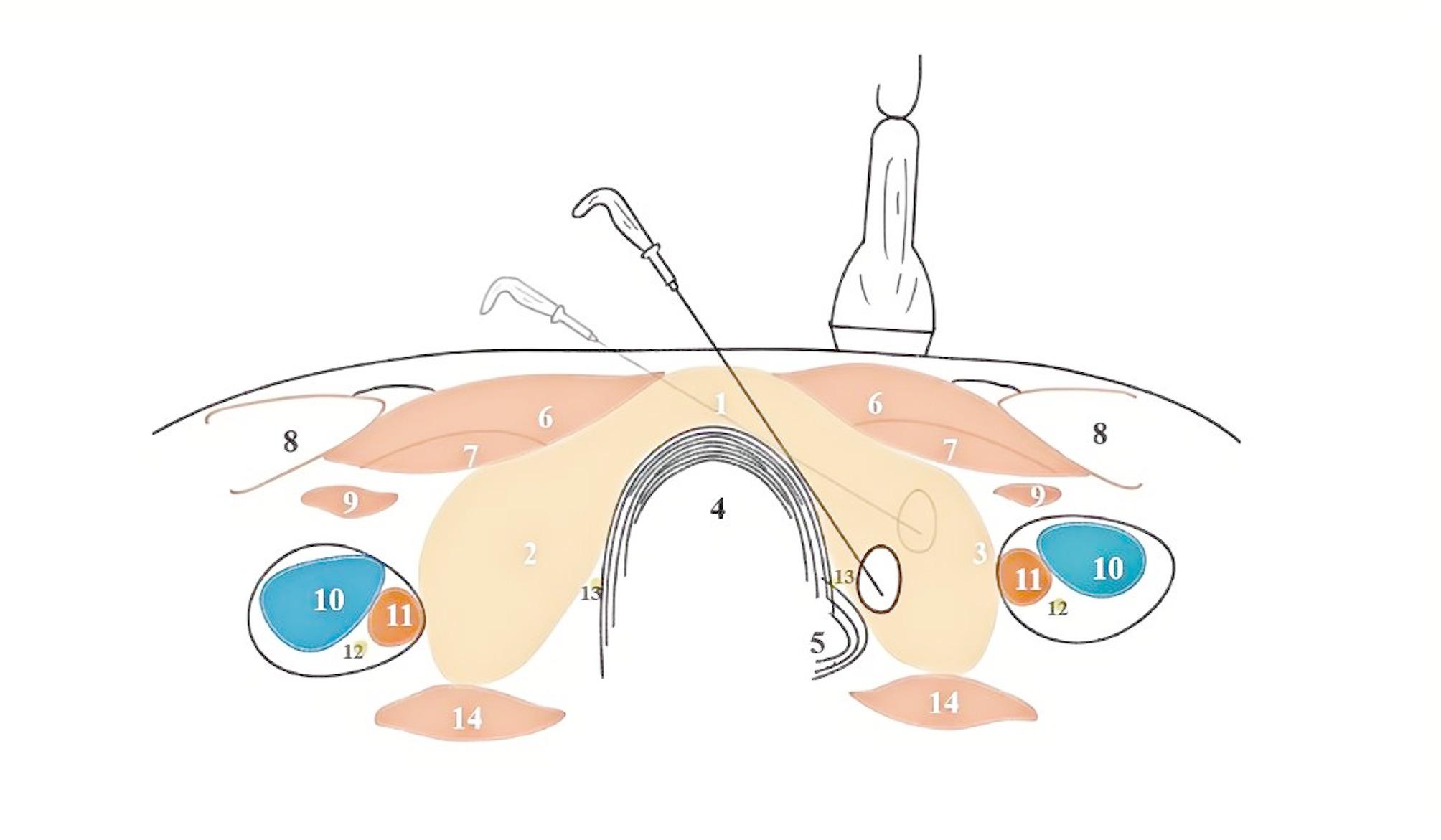
The trans-isthmic approach and alienate maneuver. The dark color indicates the initial positions of the ablation needle and nodule, whereas the light color indicates their positions after movement. 1.isthmus of thyroid 2.right lobe of thyroid 3.left lobe of thyroid 4.trachea 5.esophagus 6.sternohyoid muscle 7.sternothyroid muscle 8.sternocleidomastoid muscle 9.omohyoid muscle 10.jugular vein 11.carotid artery 12.vagus nerve 13.recurrent laryngeal nerve 14.musculus longus colli

In addition, when ablating a lateral thyroid nodule via the isthmus approach, the principle of leverage can be applied. The isthmus serves as the fulcrum, allowing the ablation needle to gently press against the lateral end, thereby generating continuous upward force on the ablation tip. This action pushes the nodule away from the “danger triangle” and the posterior chamber, enabling complete ablation of the deepest portion through moving shooting technology and reducing the overall difficulty of the procedure (Fig. 1) [67].

#### Moving shot technique

**Fig. 2:**
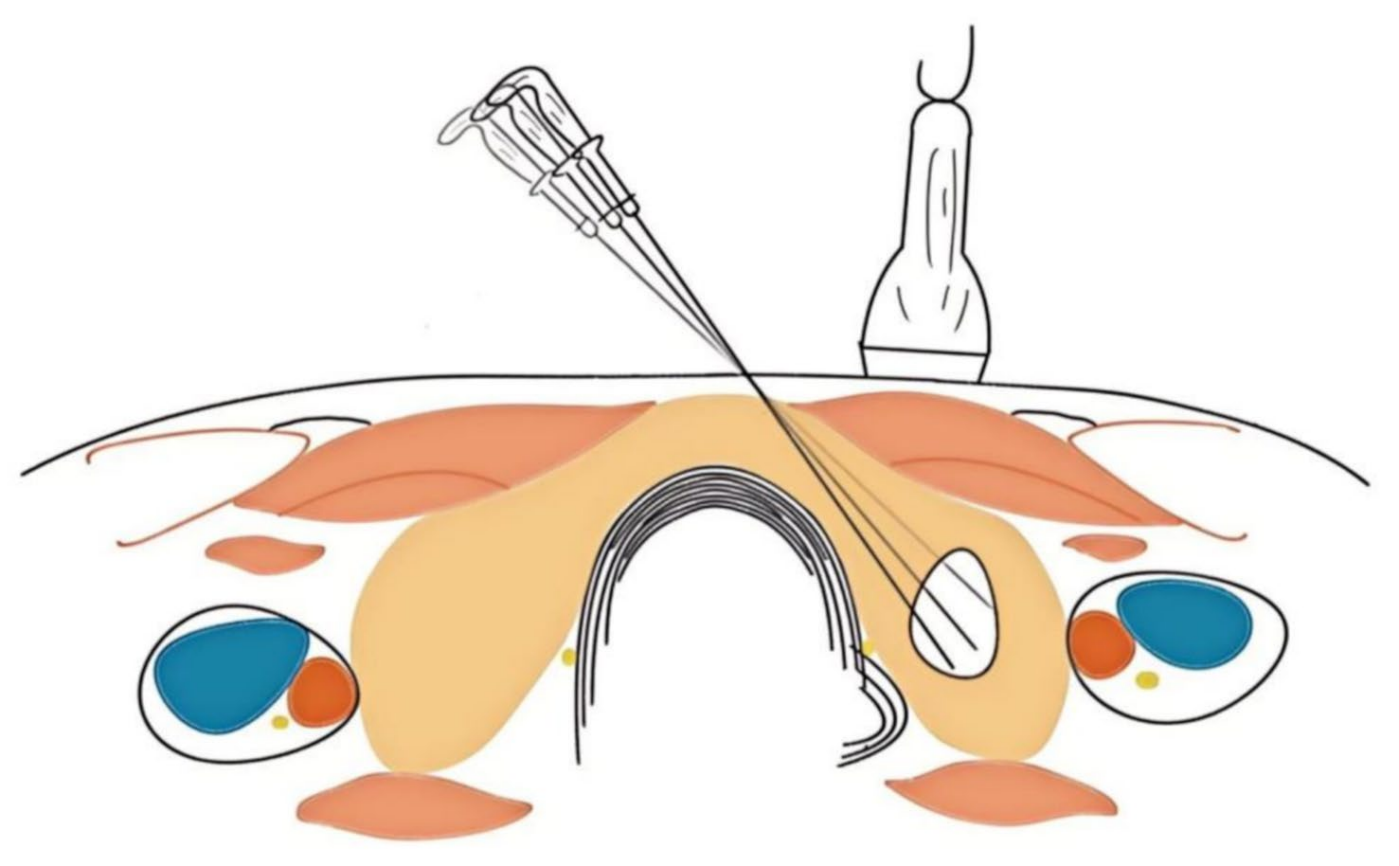
Moving Shot Technique. The transition from dark to light coloration of the ablation needle indicates the direction of its movement

Generally, the fixed needle technique is adequate for the complete ablation of small PTMC. Recently, Zhong X et al. presented compelling evidence from clinical data to support their prediction that single-applicator fixed ablation can achieve complete ablation of PTMC measuring ≤ 6.70 mm using MWA and ≤ 4.69 mm using LA [68]. For high-volume PTC, prolonged fixed-needle ablation may lead to tissue carbonization at the ablation center and delay the absorption process. Additionally, due to the irregular morphology of malignant tumors, there is a greater likelihood of residual tumor cells in the marginal areas, necessitating the use of moving shot technology. Moving shot technology refers to segment the target nodules into multiple distinct ablation units and conduct ablation in a systematic, layer-by-layer and point-by-point manner (Fig. 2). Chan WK et al. introduced a pivotal operation known as the “zigzag moving technique,” which allows to achieve larger ablation volumes in less time [67].

#### Liquid isolation

When nodules are close to critical tissues of neck, creating a liquid buffer between the target nodules and these structures can significantly reduce the risk of thermal injury during ablation. Frequently employed solutions include 5% glucose solution or 0.9% NaCl solution. A vitro study has demonstrated that during thyroid RFA treatment, the thermal insulation efficacy of the former within a 1–5 mm range is superior to that of the latter [69]. In 0.9% NaCl solution, the alternating electric field generated by RFA may induce ion agitation, leading to frictional heating. This effect increases the temperature and diminishes the thermal insulation properties of the solution. On the contrary, 5% glucose solution, which is not influenced by electrical conductivity, offers broader applicability. Due to the high fluidity of the two solutions, the ablation process requires frequent injections and intricate procedures. To address this challenge, a range of hydrogels have been developed as isolation media. These hydrogels not only provide superior thermal protection but also exhibit excellent retention properties and controllable degradability, demonstrating significant potential for clinical applications [70, 71].

**Fig. 3:**
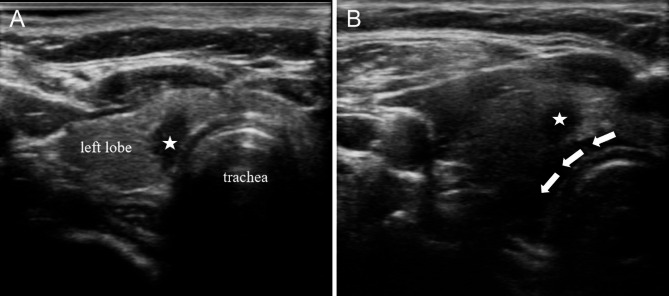
Liquid isolation in the paratracheal space. **(A)** A 41-year-old female presents with a 0.45 × 0.62 × 0.42 cm hypoechoic nodule located in the left lobe of the thyroid gland near trachea. **(B)** Liquid isolation in the paratracheal space separates the nodules from the trachea. The five-pointed star denotes the location of the nodule, while the arrow represents the direction of liquid isolation. The operator is positioned at the head of the patient

**Fig. 4:**
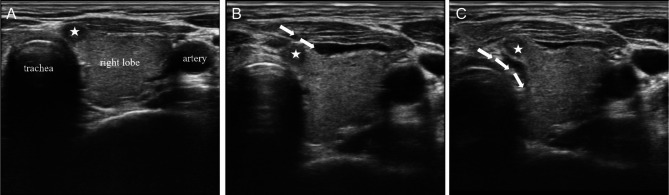
Liquid isolation in the prethyroid space and pretracheal space. **(A)** A 32-year-old female patient presented with a hypoechoic nodule measuring 0.53 × 0.47 × 0.39 cm in the right lobe of the thyroid gland, near the isthmus. **(B)** Liquid isolation in the prethyroid space separates the nodules from the anterior cervical muscle group. **(C)** Liquid isolation in the pretracheal space separates the nodules from the trachea. The five-pointed star denotes the location of the nodule, while the arrow represents the direction of liquid isolation. The operator is positioned at the head of the patient

**Fig. 5:**
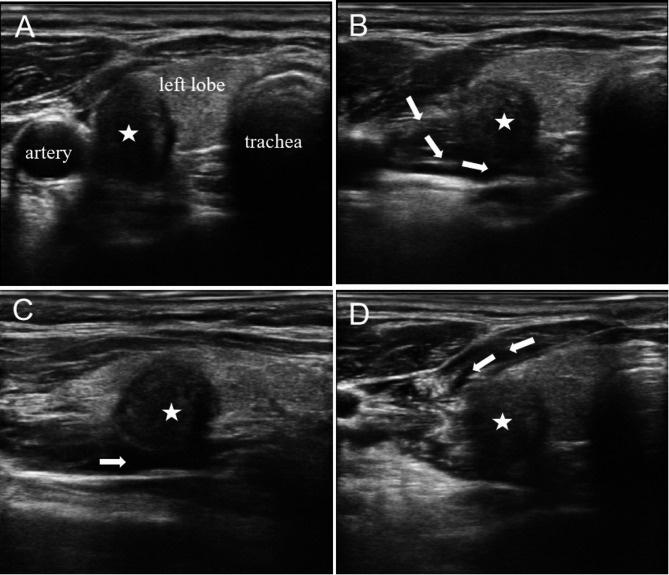
Liquid isolation in the prethyroid space the retrothyroid space. **(A)** A 51-year-old female patient was found to have a 1.1 × 1.3 × 1.3 cm hypoechoic nodule in the left lobe of the thyroid gland. **(B)** Saline injection extends from the lateral aspect of the thyroid gland to the retrothyroid space, creating an isolation zone. **(C)** Viewed longitudinally, the liquid isolation distinctly segregates the nodule from the dorsal membrane of thyroid. **(D)** Liquid isolation in the prethyroid space separates the nodules from the anterior cervical muscle group. The five-pointed star denotes the location of the nodule, while the arrow represents the direction of liquid isolation. The operator is positioned at the head of the patient

Based on the location of the nodule, the operator can devise personalized fluid isolation strategies. It is recommended to employ fascial-based fluid isolation techniques, ensuring that the lesion is separated from surrounding critical structures by more than 0.5 cm. These spaces include: (1) the prethyroid space, the area between the prethyroid membrane and the anterior cervical muscle group; (2) the retrothyroid space, the area between the dorsal membrane of the thyroid gland and the longus colli muscle as well as the carotid sheath; (3) the paratracheal space, the area between the medial thyroid membrane and the trachea, esophagus, and recurrent laryngeal nerve [72]. In general, if the nodule is small and adjacent to a single space, fluid isolation of only one fascial space is enough (Fig. 3). If the nodules are large and adjacent to multiple spaces, fluid isolation of multiple fascial spaces is necessary (Fig. 4 and Fig. 5) [73]. For lesions adjacent to the “danger triangle” region, it is advisable to continue injecting appropriate volume of isolation fluid during ablation. This practice helps to lower the temperature in the ablation zone and mitigates the risk of residual heat damaging the recurrent laryngeal nerve.

### Stringent postoperative follow-up

#### Choice of postoperative evaluation and follow-up

In clinical practice, CEUS is routinely conducted immediately following ablation to assess the completeness of the procedure. If the non-enhanced region extends more than 2 mm beyond the margin of the original lesion, it is deemed that the ablation has been successfully completed [74]. Otherwise, additional ablation may be warranted.

There is currently no standardized protocol for follow-up and management after ablation. The majority of studies have relied solely on conventional US or CEUS for monitoring. On ultrasound imaging, the ablation area of the original lesion typically appears as a hypoechoic or heterogeneous region. Because malignant lesions also present as hypoechoic areas, it is insufficient to differentiate between residual tumor tissue and the ablated region. Only a limited number of studies utilized biopsy follow-up [16, 75]. Compared with ultrasound follow-up, the detection rate of incomplete ablation was notably higher using biopsy follow-up, thereby highlighting the limitations of follow-up based on the imaging methods.

Few studies has been conducted to predict the risk of recurrence in patients with PTC following ablation therapy. Sim JS et al. were the first to introduce the initial ablation ratio (IAR) as a quantitative metric for predicting the efficacy of RFA in the treatment of benign thyroid nodules [76]. IAR is defined as the ratio of ablation area measured by CEUS on the day or the next day of ablation, to the preoperative ablation volume. Ren Y et al. utilized the IAR as a predictive indicator for the recurrence of MWA treatment in low-risk PTMC. They found that the prognosis of patients with an IAR of 15 or higher was significantly improved compared to those with an IAR of less than 15. Consequently, a larger ablation area was associated with a reduced risk of recurrence [77]. Furthermore, Li X et al. developed a response-to-ablation system that classifies patients into three categories based on their response to therapy at the 1-year follow-up: (1) A complete response was defined as complete disappearance on US or incomplete disappearance on US with benign CNB/FNA. (2) A indeterminate response was defined as incomplete disappearance without CNB/FNA or suspicious nodule or lymph node on US without confirmed pathology. (3) An incomplete response was defined as FNA/CNB confirmed for persistent PTMC in the ablation area, newly found PTMC, or LNM. This classification system demonstrated significant efficacy in predicting the risk of local recurrence after RFA treatment for PTMC [78].

#### Thyroid stimulating hormone (TSH) suppression therapy

For patients with thyroid cancer who have undergone lobectomy, levothyroxine hormone replacement therapy is frequently necessary to maintain optimal TSH levels. This therapeutic approach plays a crucial role in the postoperative management and long-term care of these patients. The 2015 American Thyroid Association guidelines recommend that for patients with low-risk thyroid cancer, TSH levels should be maintained within the range of 0.5-2 mIU/L during follow-up after lobectomy of thyroid [79]. As thermal ablation selectively targets and destroys only the thyroid tissue in the vicinity of the cancer area, the remaining healthy thyroid tissue retains its capacity to maintain TSH levels within the normal range, so routine levothyroxine hormone therapy is not typically required. In a study of 516 low-risk PTC patients treated with RFA, propensity score-matched analyses demonstrated that local tumor progression and disease-free survival were comparable between groups with higher TSH levels (TSH > 2 mU/L) and lower TSH levels (TSH ≤ 2 mU/L). So, the optimal TSH level is recommended at the euthyroid range after thermal ablation therapy of low-risk PTC [80]. A recent large-scale, real-world, retrospective study involving 11,140 patients with low-risk PTC revealed that postoperative TSH levels were not significantly associated with tumor recurrence [81]. Currently, it is widely accepted that TSH suppression therapy is not required after thermal ablation of low-risk PTC, provided that normal thyroid function is maintained.

## Conclusion

Thermal ablation has emerged as a promising minimally invasive treatment for PTC, favored by patients for its minimal invasiveness, low tissue damage, and repeatability. However, the issue of postoperative tumor progression remains a concern. To minimize the risk of tumor progression, there are several key recommendations for thermal ablation of PTC. (1) Thermal ablation therapy for PTC requires strict adherence to indications, including a maximum tumor diameter ≤ 10 mm. (2) The ablation zone must extend ≥ 2 mm beyond the tumor boundary to ensure complete coverage. (3) CEUS is required for pre-procedural planning and immediate post-procedural assessment. (4) When suboptimal involution or abnormal internal blood flow signals are detected by US or CEUS during follow-up, FNAB or CNB should be performed. (5) Procedures should only be performed at high-volume centers by operators with certified expertise. While these strict criteria are essential for current practice to minimize progression risk, the potential for expanding indications to include larger tumors remains a critical topic requiring further investigation.

## Data Availability

No datasets were generated or analysed during the current study.

## Abbreviations

PTC: Papillary thyroid carcinoma
US: Ultrasound
RFA: Radiofrequency ablation
MWA: Microwave ablation
PTMC: Papillary thyroid microcarcinoma
LNM: Lymph node metastasis
CEUS: Contrast-enhanced ultrasound
FNAB: Fine-needle aspiration biopsy
CNB: Core needle biopsy
CT: Computed tomography
MRI: Magnetic resonance imaging
LCEUS: Lymphatic contrast-enhanced ultrasound
TERT: Telomerase reverse transcriptase
FNA-Tg: Thyroglobulin in fine needle aspiration
TSH: Thyroid stimulating hormone
